# Cholelithiasis in Thalassemia Major Patients: A Report from the South-East of Iran

**Published:** 2018-04-01

**Authors:** Iraj Shahramian, Razieh Behzadmehr, Mahdi Afshari, Atefeh Allahdadi, Mojtaba Delaramnasab, Ali Bazi

**Affiliations:** 1 Pediatric Digestive and Hepatic Research Center, Zabol University of Medical Sciences, Zabol, Iran; 2 Department of Radiology, Zabol University of Medical Sciences, Zabol, Iran; 3 Department of Community Medicine, Zabol University of Medical Sciences, Zabol, Iran; 4 Clinical Research Development Unit, Amir-Al-Momenin Hospital, Zabol University of Medical Sciences, Zabol, Iran

**Keywords:** Cholelithiasis, Gallstone, Thalassemia

## Abstract

**Background: **Cholelithiasis and its predisposing factors are less characterized in thalassemia syndromes. In the present study, we assessed the prevalence of gallstones and related-risk factors among thalassemia major (TM) patients in south-east of Iran.

**Materials and Methods: **The patients were recruited form a single center in Zabol city, south-east of Iran. Demographic and clinical information were retrieved from medical histories. Abdominal ultrasonography was performed to scrutinize gallstones and organ dimensions of liver, spleen, gallbladder and kidney.

**Results: **The study participants (n=127) consisted of 50 (39.4%) males and 77 (60.6%) females. The mean age of the patients was 15.2±7.9 years**. **Cholelithiasis was observed in 11 (8.7%) patients. Cholelithiasis was significantly associated with age (*P*=0.002) and splenectomy (*P*=0.001). The patients with cholelithiasis received a significantly higher blood volume than patients without cholelithiasis (546±108.7 ml and 425.1±134.7 ml, respectively, *P*=0.007). There were significant differences between cholelithiasis and non- cholelithiasis TM patients regarding the length of right and left liver lobes (*P*=0.001), as well as the length of gallbladder (*P*=0.006). Ferritin level was not associated with cholelithiasis in our patients. In multivariate analysis, age older than 15 (OR=10.4, 95% CI: 1.2-86.3, *P*=0.02) and 30 years old (OR=42.6, 95% CI: 2.9-613, *P*=0.006), and splenectomy (OR=8.7, 95% CI: 2.1-35.4, *P*=0.002) were significant risk factors for cholelithiasis.

**Conclusion: **Cholelithiasis is a relatively common complication among TM patients in our region. The most prominent risk factors of cholelithiasis were advanced age, splenectomy and large-volume blood transfusion.

## Introduction

 Thalassemia is the most common monogenic disorder and a global health issue. This syndrome is highly frequent in the Middle East countries including Iran. Thalassemia syndromes arise from deactivating mutations or deletions in alpha or beta globin genes  ^1^^,^^2^ . Depending on the severity of the abrogation of globin chains output, thalassemia is classified into three main phenotypic categories: thalassemia minor, thalassemia intermediate (TI) and thalassemia major (TM)^   3 ^. Among these clinically important forms, TI and TM are dependent on therapeutic interventions particularly regular transfusions.

Although blood transfusions have resulted in magnificent improvement in the life expectancy of TM patients, secondary transfusion-related organ failure is a concerning issue among these patients. Patients with TM need regular blood transfusions at two- to four-week intervals for ensuring sufficient tissue oxygenation. Transfusion -related hemosiderosis is considered as the main mechanism responsible for organ dysfunction in TM patients. The most common complications include cardiomyopathy, cholelithiasis, osteoporosis and endocrinopathies ^   4 ^. Despite notable achievements, current therapeutic protocols of TM are sufficient neither for prevention nor for management of these complications. 

Cholelithiasis is defined by the presence of radiological evidence of gallstones  ^5^^,^^6^ . This complication has been reported in 10-57% of TM patients  ^5^^,^^7^^-^^10^ . Pathogenesis of cholelithiasis in TM is multifactorial. The main contributing factor is deemed to be precipitation of bilirubin in the bile as a result of increased hemolysis ^   11 ^. Iron deposition within gallbladder is also involved in the development of cholelithiasis ^   12 ^. The role of ineffective erythropoiesis has also been suggested in the formation of gallstones in TM  ^13^^,^^14^ . Regarding inconsistencies over the pathogenesis and contributing factors in cholelithiasis, there is a need for further evaluation of potential mechanisms predisposing to cholelithiasis in TM. 

Sistan and Baluchistan province in the south-east of Iran is a region with high frequency of thalassemia in Iran  ^15^^,^^16^ . Thalassemia patients in this province are unique regarding ethnic heterogenicity and genetic features. There were no previous studies on the prevalence and risk factors associated with cholelithiasis in TM in this region. In the present study, we evaluated 127 TM patients in Zabol city, the second most populated city in the province, regarding the prevalence of cholelithiasis and its risk factors.

## MATERIALS AND METHODS

 The current cross-sectional study was performed in Zabol city, Sistan and Baluchestan province of Iran from April to September 2016. The study population included 127 TM patients in the only care center located in the city. The patients were under transfusion every 2-4 weeks. They were included into the study based on a targeted sampling method. The patients or their parents were requested to fill out an informed consent. Demographic and clinical data were extracted from medical records available at the center.

Abdominal ultrasonography was performed for diagnosis of gallstones. Longitudinal and transverse planes were performed for seeking evidence of gallstones in hepatobiliary tree. Ultrasonography was also performed for determination of liver, spleen and kidney dimensions.

Based on spleen length and upper limit of spleen size previously reported by Rosenberg et al.^   17 ^, non-splenectomized patients were categorized into groups of normal size, mild (<2 cm above upper limit) , moderate (2-4 cm above upper limit) and severe splenomegaly (>4 cm above upper limit) ^   9 ^.

Statistical analysis was performed with SPSS Version 19.0 statistic software package. Normality of data was assessed using Shapiro–Wilk test. Frequencies and descriptive statistics were used for presenting appropriate variables. Univariate analysis included Chi-square and independent-samples student’s t-test. In multivariate analysis, logistic regression was used to predict risk factors for cholelithiasis.

## Results

 The study participants (n=127) consisted of 50 (39.4%) males and 77 (60.6%) females. The study cohort comprised 35(27.6%) splenectomized patients. Mild, moderate and severe splenomegaly were observed in 18 (19.6%), 8 (8.7%), and 5 (5.4%) patients, respectively. The mean age of the patients was 15.2±7.9 years. Mean hemoglobin was measured 9.2±0.9 g/dl. The patients had mean ferritin level of 2846.3±1752 ng/ml (range of 114-7750). The patients were transfused with mean blood volume of 434.8±136.5 ml every 2-4 weeks. Ultra-sonographic findings for dimensions of liver, spleen, gallbladder and kidney are shown in Table 1.

**Table 1:** Ultrasonography findings in 127 thalassemia major patients

Ultrasonography findings (mm)	Minimum	Maximum	Mean	Standard deviation
**Right lobe liver ** **length**	19	51	33.3	14
**Right lobe liver ** **width**	5	18	11	2.6
**Right lobe liver ** **depth**	4	16	8.5	2.2
**Left lobe liver ** **length**	17	50	31.1	7.6
**Left lobe liver ** **width**	6	18	10.6	2.5
**Left lobe liver ** **depth**	4	18	8.2	2.2
**Gallbladder length**	15	109	59.8	21.2
**Gallbladder width**	8	28	17.1	6
**Spleen length**	70	220	109.6	29.8
**Spleen width**	32	190	99.5	26.9
**Spleen depth**	22	100	41.1	12.1
**Right kidney ** **length**	70	137	98.6	13.6
**Right kidney ** **width**	28	64	46.3	7
**Right kidney ** **depth**	24	55	36.6	5.7
**Left kidney length**	64	172	100.3	15.6
**Left kidney width**	24	80	44.9	7.9
**Left kidney depth**	17	69	35.7	7.3

Cholelithiasis was observed in 11 (8.7%) of patients. For the patients diagnosed with cholelithiasis in sonographic evaluation, mean diameter of gallstones was 7.7±2.7 mm (range of 3-11 mm). This value showed no differences regarding gender, splenectomy, spleen size, ferritin, or mean blood volume received per transfusion.

Splenectomy was significantly associated with cholelithiasis (*P*=0.001). However, there was no significant difference in frequency of cholelithiasis regarding different genders or different ferritin categories (Table 2). Patients with cholelithiasis had significantly higher mean age (24.1±8.4) than patients without cholelithiasis (14.3±7.3, *P*<0.0001). Also, patients transfused with a higher mean blood volume showed higher ratio of cholelithiasis (Table 3).

**Table 2:** Comparison of demographic and clinical features of TM patients with and without cholelithiasis

Parameters	Cholelithiasi s N=11		Non- Cholelithiasi s N=116		P
	N	%	N	%	
**Gender**	Male	6	54.5	44	37.9	0.2*
	Female	5	45.5	72	62.1	
**Age ** **Categories ** **(year)**	<15	1	9	64	55.2	0.002
	15-30	8	72.8	49	42.3	
	>30	2	18.2	3	2.5	
**Splenectom** **y**	Yes	8	72.2	27	23.2	0.001
	No	3	27.3	89	76.8	
**Spleen size**	Normal	2	66.6	59	66.2	0.1
	Mild splenomegal y	0	0	18	20.3	
	Moderate splenomegaly y	0	0	8	8.9	
	Severe splenomegaly y	1	33.4	4	4.6	
**Transfused ** **blood per ** **occasion ** **(ml)**	<250	0	0	16	13.7	0.07
	250-500	3	27.2	60	51.7	
	>500	8	72.8	40	34.6	
**Ferritin ** **(ng/ml)**	<1500	2	18.2	31	27.2	0.6
	1500-2500	3	27.3	30	25.7	
	>2500	6	54.5	55	47.1	

**Table 3:** Comparison of mean values of selected variables in TM patients diagnosed with cholelithiasis and with non-cholelithiasis patients

Parameters	Cholelithiasis N=11		Non- Cholelithiasis N=116		P
	Mean	SD	Mean	SD	
**Age (years)** **Hemoglobin ** **(g/dl)**	24.1 9.5	8.4 0.9	14.3 9.2	7.3 0.9	<0.001 0.3
**Ferritin ** **(ng/ml)**	4090.6	2653.8	2752.4	1645.3	0.2
**Received ** **blood per ** **transfusion ** **(ml)**	546	108.7	425.1	134.7	0.007

There were significant differences in means of liver right lobe length (*P*=0.001), liver right lobe depth (*P*=0.02), liver left lobe length (*P*<0.0001) and gallbladder length ((*P*=0.006) between the patients with and without cholelithiasis. Table 4 shows ultrasonography-derived dimensions of liver, gallbladder, spleen, and kidney in the study participants. Logistic regression analysis revealed the advanced age and splenectomy as the most prominent risk factors for cholelithiasis in our patients (Table 5).

**Table 4:** Comparison of ultrasonography derived dimensions of liver, spleen, gallbladder and kidney in TM patients with and without cholelithiasis

Ultrasonography findings (mm)	Cholelithiasis N=11		Non- Cholelithiasis N=116		*P*
	**Mean**	**SD**	**Mean**	**SD**	
**Right lobe liver ** **length**	41.1	6.3	32.6	7.4	0.001
**Right lobe liver ** **width**	12.4	2.5	10.9	2.6	0.1
**Right lobe liver ** **depth**	9.9	2.6	8.4	2	0.02
**Left lobe liver ** **length**	39.3	6.9	30.3	7.3	<0.0001
**Left lobe liver ** **width**	11.9	2.8	10.5	2.5	0.1
**Left lobe liver ** **depth**	9.5	3.2	8.1	2.1	0.06
**Gallbladder length**	31.6	14.4	65.8	17.3	0.006
**Gallbladder width**	16.3	7.6	17.3	5.9	0.8
**Spleen length**	142.6	64.4	108.5	28	0.4
**Spleen width**	122	42.3	98.8	26.3	0.1
**Spleen depth**	58.3	36.1	40.5	10.6	0.4
**Right kidney ** **length**	108.3	10.2	97.8	13.6	0.02
**Right kidney width**	51.8	6.9	45.8	6.9	0.01
**Right kidney depth**	39.1	6.2	36.4	5.6	0.1
**Left kidney length**	110.7	14.8	99.4	15.4	0.02
**Left kidney width**	53.3	12.7	44.1	7	0.05
**Left kidney depth**	42	11.4	35.1	6.6	0.004

**Table 5:** Risk factor for cholelithiasis in TM patients based on logistic regression analysis

**Parameters**		**Non-corrected**		**P**	**Corrected**		**P**
		**OR**	**95% I**		**OR**	**95% CI**	
Age (years)	<15	Reference			Reference		
	15-30	10.4	1.2-86.3	0.02	5	0.4-52.8	0.1
	>30	42.6	2.9-613	0.006	15.8	0.8-305.6	0.04
Splenectomy	No	Reference			Reference		
	Yes	8.7	2.1-35.4	0.002	3.6	0.7-17.7	0.1

## Discussion

 Regarding the high rate of hemolysis and ineffective erythropoiesis, TM patients are susceptible to cholelithiasis, which can indicate the presence of gallstones in gallbladder. In the current study, 116 TM patients from south-east of Iran were evaluated for evidence of cholelithiasis. For this purpose, the patients were undergone abdominal ultrasonography which is considered as a sensitive method for detecting cholelithiasis. Overall, 11/127 (8.7%) patients were diagnosed with cholelithiasis. Cholelithiasis was also reported in 29 (31.5%) among 92 TI patients in Shiraz, Iran ^        13 ^. In another study in North of Iran, cholelithiasis was reported in 17 (23.6%) out of 72 thalassemia patients ^   14 ^. Cholelithiasis was also described in 20.3% of TM and 57.1% of TI patients in Iran ^   10 ^. The prevalence of cholelithiasis in thalassemia carriers was reported as 20.3% ^   18 ^. Lotfi et al. evaluated 153 β-TM and 52 TI patients and reported cholelithiasis in 15 (9.8%) patients ^   9 ^. In a large population study on TI patients in Iran, 153 subjects were evaluated for thalassemia- related complications and cholelithiasis was reported in 25.5% of the participants^19^. This is while cholelithiasis has been reported in 0.1-0.3% of general pediatric population (20). This result was obtained while cholelithiasis was reported in 30-56% of TM patients in Italy  ^21^^, ^^22^ , 21.6% in the US ^        23 ^ and 6- 18% in Egypt  ^24^^, ^^25^ . Compared to previous studies, lower ratio of TM patients with cholelithiasis was identified in the present study.

The relatively wide variation reported in different studies may reflect variations in patients’ characteristics regarding demographic, transfusion history or genetic propensities  ^21^^, ^^26^^, ^^27^ . Gilbert syndrome mutation has been the most dominant genetic contributor to formation of gallstones in thalassemia syndrome  ^18^^, ^^28^ . The rate of hemolysis also affects progression of cholelithiasis in TM. An elevated serum bilirubin level as a result of chronic hemolysis may also be involved in the formation of gallstones. The role of hemolysis, however, has been questioned by the fact that many TM patients with severe hemolysis do not form gallstones ^        22 ^. Despite the milder clinical severity and lower frequency of transfusions, the ratio of cholelithiasis has been higher in TI patients than TM ^   10 ^. This may highlight the potential role of disease-specific features in promoting gallbladder pathology in TM. 

The role of nutritional factors must be considered in propensity to cholelithiasis ^   29 ^. Previous studies indicated higher risk of gallstones in individuals consuming moderate-high fat diets compared to those following low-fat diets ^   29 ^. *Cholesterol as the major *constituent accounts for most *gallstones* in patients with cholelithiasis ^        30 ^. On the other hand, abnormal mechanical properties of gallbladder in TM patients may further contribute to development of gallstones. Gallbladders of TM patients have shown lower motility and contractibility, longer emptying times and higher residual and fasting volumes compared to healthy counterparts  ^22^^-^^24^ . Further studies are needed to reveal the potential role of gallbladder functional abnormalities in pathogenesis of cholelithiasis in TM.

Among the studied clinical and demographic features, the presence of cholelithiasis was significantly related to older age, splenectomy and transfusion magnitude. The role of splenectomy in predisposition to cholelithiasis has been described previously  ^9^^,^^13^^,^^19^ . The ratio of thalassemia patients (68.4%) with cholelithiasis splenectomized in the study conducted by Lotfi et al ^9^ was close to the ratio reported in present study (72.2%). Generally, advanced age has been noted as a significant risk factor for cholelithiasis among TM patients  ^9^^,^^19^^,^^25^^,^^28^ . In the current study, only one patient under 15 years of age showed cholelithiasis and the rest showed it in the second and third decades of life. Similarly, we found that TM patients with cholelithiasis had significantly higher mean age (24.1±8.4 years old) than those without cholelithiasis (14.3±7.3 years old, *P*<0.001). TM patients with cholelithiasis require large-volume blood transfusion  ^9^^,^^25^ . Based on the findings of the current study, TM patients with cholelithiasis received higher mean blood volume (546±108.7 ml) than patients without cholelithiasis (425.1±134.7 ml, *P*=0.007). Other predisposing factors of cholelithiasis in thalassemia syndromes included coexistence of gilbert syndrome and TI phenotype^28^. Higher pretransfusion hemoglobin level has also been noted as a risk factor for cholelithiasis in TM^25^. However, there was no significant difference between pretransfusion hemoglobin level of TM patients with and without cholelithiasis in our study. Although it has been suggested that female gender may be a risk factor for gallstones in general population ^        30 ^, we found no correlation between gender and cholelithiasis in our study which is in agreement with previous reports ^   9 ^. Totally, splenectomy, advanced age and large-volume blood transfusion seem to represent major risk factors for cholelithiasis in thalassemia patients. The role of these acquired parameters may be modified by genetic propensity of patients. 

## CONCLUSION

 According to our results, gallstone is a relatively common complication in TM patients. The most significant predictors of cholelithiasis were advanced age, splenectomy and larger volume of blood transfusion. However, it seems that functional properties of gallbladder or genetic determinants may contribute to the risk of cholelithiasis. It is recommended to routinely evaluate TM patients, especially older patients, for the presence of cholelithiasis.
